# Adolescent and parental views on weight and weight management: a qualitative study

**DOI:** 10.1136/archdischild-2024-327306

**Published:** 2025-02-06

**Authors:** Melissa Little, Susan A. Jebb, Paul Aveyard

**Affiliations:** 1 Nuffield Department of Primary Care Health Sciences, University of Oxford, Oxford, UK

**Keywords:** adolescent health, obesity, paediatrics, qualitative research

## Abstract

**ABSTRACT:**

**Objective:**

To examine the views of adolescents with excess weight and parents of adolescents with excess weight towards weight and weight management.

**Design:**

A qualitative study design using semi-structured phone interviews. The interviews were recorded, transcribed verbatim and analysed using reflexive thematic analysis to interpret the data.

**Settings:**

Parents and adolescents based in the UK and recruited through schools, social media and youth centres.

**Participants:**

Ten parents and 16 adolescents, including four linked parent-child dyads.

**Results:**

Both parents and adolescents reported that adolescents felt societal pressure to achieve an ‘ideal body’ and that this pressure was the primary motivator for weight loss. All interviewed parents perceived themselves as overweight; however, those who had minimal weight-based shame were more likely to normalise weight discussions, reducing weight shame in their children. Adolescents preferred parents to display healthy behaviours rather than initiate weight-based discussions; however, they wanted to feel supported if the conversations were self-initiated.

**Conclusion:**

Weight is a sensitive issue in intergenerational relationships, primarily driven by feelings of shame. Adolescents feel supported by a ‘show not tell’ approach from parents, which is more likely in families with less shame. While guidelines encourage clinicians to initiate weight-related conversations, this may not be welcomed by adolescents, although they do want confidence that they could access weight management support if they choose to seek help.

**WHAT IS ALREADY KNOWN ON THIS TOPIC**

- Obesity in adolescents is increasing worldwide, and current treatment options show minimal long-term success.
- Adolescence is a period that is vulnerable to the onset of disordered eating behaviours, leading to conflicting views on whether weight reduction behaviours are appropriate.
- Parents are reluctant to discuss weight or engage in treatment interventions for fear of the ‘triggering effect’ these discussions may have.

**WHAT THIS STUDY ADDS**

- Weight pressure is common among young people and is the primary motivator for weight loss in the age group.
- Parental feelings towards their own weight seems to influence how they approach their child’s weight.
- Adolescents prefer to avoid weight-based discussions unless self-initiated, in which case they want to feel heard and offered support.

**HOW THIS STUDY MIGHT AFFECT RESEARCH, PRACTICE OR POLICY**

- Counter to national guidance, proactive interventions by clinicians may not be welcomed by adolescents who would prefer to raise the topic themselves. Additionally, the avoidance of intergenerational weight-based discussions suggests the need for weight support that does not require a parental proxy for access.

Obesity in adolescents is increasing worldwide.^1^ Overweight and obesity in adolescents are clinically defined as having a body mass index (BMI) greater than or equal to the 91st and 98th centile, respectively, on the UK 1990 growth reference charts.^2^ Excess weight in in this age group is associated with an increased risk of physical, mental and psychosocial complications in the short and long term.^3^ For adolescents who are clinically overweight, guidelines recommend maintenance or reduction in BMI z-score to reduce future health risks,^4^ however weight treatment interventions in this age group have limited long-term success,^5^ suffering low uptake and high attrition rates.^5^,^7^

Adolescence is also a period that is vulnerable to the onset of disordered eating behaviours^8^ and some evidence suggests that adolescent weight control behaviours may lead to adverse mental and physical health consequences.^9^ Additionally, there is a shared apprehension among both parents and adolescents of weight-based discussions in both clinical and non-clinical settings and the potential ‘triggering effect’ these may have on mental health.^10 11^ Yet, adolescents living with obesity express a desire to lose weight,^12^ and to best support them, it is crucial to illicit their views on the type of support they require. Thus far, research directly involving adolescents’ views on weight and weight management is scarce, with research looking at the parent-child dyad even more limited.

The aim of this study is to understand these concerns from both adolescents’ and parents’ perspectives. These findings would have implications for whether and what support adolescents need to achieve a healthier weight status.

## Methods

### Design, setting and participants

The study recruited from England and Wales and young people were eligible if they were aged 13–17 years and self-identified as being above a healthy weight. Parents were eligible if they had a child aged 13–17 years who they identified as being above a healthy weight. The original intention was to recruit linked parent-child dyads; however, as weight is often a sensitive topic to discuss, recruitment was challenging, and we therefore switched to include non-linked participants. Recruitment challenges also meant purposive sampling was not feasible and therefore we reverted to a convenience and snowball sampling approach. Sample size was determined through data saturation, meaning that interviews ceased once enough in-depth data were obtained to support robust themes and subthemes and additional data did not yield new conclusions.^13^

Participants were recruited through youth groups, secondary schools and social media including, Twitter, Netmums and Nextdoor. Before the interviews took place, potential participants were sent a participant information sheet (PIS) to read through and ask the researcher questions. For participants under the age of 16 years, the PIS was sent to their parent or carer with an additional simplified PIS sent to the potential participant themselves. Participants were then contacted to ascertain their willingness to take part in the study and verbal consent was obtained. For participants under the age of 16 years, parents consented and participants assented. A £20 retail voucher was given to all participants as compensation for their time.

### Data collection

Data were collected using semi-structured interviews conducted by ML between February 2022 and March 2023. Interview guides were developed prior to the study with different guides for parents and for adolescents. Both the parent and adolescent interviews covered topics such as feelings towards weight, the effect of weight on the parent/child relationship and the weight management journey of the young person. Interviews were flexible and content was iteratively adapted based on previous interviews. The original interview guides are available in online supplemental appendix 1.

All interviews were conducted over the telephone, audio recorded and immediately uploaded onto a secure drive on the University servers. ML took field notes after each interview to capture any initial thoughts on the interview or ways and reasons that it may have diverted from the original interview guide. These initial reflections were analysed with interview transcripts to form background to the interview.

### Data analysis

Data were analysed by ML using reflexive thematic analysis to interpret meaning and identify themes. The approach is grounded in the literature, while also allowing for an inductive, interpretive and reflexive methodology.^14^ As a white female and a mother, ML had potential biases around parenting, gender and dieting and, and as a dietitian and postgraduate student had contextual pre-existing clinical knowledge of obesity. ML sought to bear in mind her own contextual knowledge and potential biases, and we felt it was important not to predefine the themes to minimise personal bias. The ontological and epistemological positioning followed a subtle realism approach as defined by Pope and Mays.^15^

For the initial analysis, the interviews were listened to repeatedly for familiarity, and notes made on each. The interviews were transcribed verbatim by an external transcription service and the transcriptions were re-read to check for errors and increase familiarity. Data were uploaded to NVivo and coded by ML, using a reflexive process. As no relationship was established prior to the interview, notes were made of any points in the interview where the power differential between the interviewee and the interviewer may have affected the discussion. The result of the coding was used to create patterns of shared meaning in the interviews, which were grouped into four themes.

The study was conducted and reported in accordance with the Consolidated criteria for Reporting Qualitative research guidelines.^16^

## Results

Twenty-six participants were enrolled; 10 parents and 16 young people. A further three young people and one parent expressed interest but did not take part in the study. Of the participants, there were four linked parent-child dyads. Table 1 shows the demographic characteristics of the participants.

**Table 1:** Characteristics of study participants

	N	Self-identified ethnicity (%)	Deprivation quintile*	Gender (n)	Age in years (mean; range)
Parents	10	70% White British 10% Black African 10% Black Caribbean 10% White other	Mean=3.3 SD=1.3	Female (10)	n/a
Adolescents	16	50% White British 19% Black African 19% South Asian 6% White other 6% Asian other	Mean=3.3 SD=0.95	Female (12) Male (4)	16.3 (14–17)
Linked dyads	4	50% White British 25% Black African 25% White other	Mean=2.75 SD=0.5	Parent: Female (4) Child: Female (3), male (1)	Parent: n/a Child: 15.8 (14–17)

* Level of deprivation based on English Index of Multiple Deprivation, where 1 is the most deprived and 5 is the least deprived quintile.

n/a, not available.

The minimum interview length was 15 min and 43 s, and the maximum was 37 min and 45 s. The mean for the parent interviews was 25 min and 45 s, and the mean for the young people was 24 min and 27 s.

The following four themes were interpreted through analysis of the data. Participant quotations are provided in table 2.

**Table 2:** Illustrative quotations of themes

1. Weight pressure as a function of societal norms	“it comes from social media and like a lot of the people like get like most famous on social media and on YouTube are always like the really skinny people”. (adolescent, female, 13A) “they go on this social media and they see all these pictures and they believe they’ve got to be this certain figure, this certain look and she’s not going to be that”. (mum, female, 6B) "a woman’s life is from the moment you become aware of it thinking about your weight and it makes me so angry and so sick that that’s all we ever think about. So, I imagine it’s definitely started for her”. (mum, female, 4B) “I feel like there’s a social pressure to have an eating disorder, like, as well, as like over COVID and things it kind of became a trend to have an eating disorder and then I think that pushed a lot of people into it and then all like these discover disordered eating habits and then try and make them out as it they are healthy and push them on to their friends”. (adolescent, female, 13A) "to be liked or to be popular, you have to be like that. You have to be attractive. You have to be slim. You have to be able to fit in these clothes because that’s what’s popular. And that’s what people like”. (adolescent, female, 8A) "I feel like its more accepting towards all the different types of body, but there is that kind of criticism where you are treated different if you don’t look like the type of body that everyone would prefer you look like”. (adolescent, female, 7A)
2. The parent-child relationship	“when we talk to these kind of professionals I always feel bad because I think its my fault”. (parent, female, 8B) “opening up the conversation up at a younger age, 100%, because I think if it was a conversation that was held from when you were are 11 and 12 people would feel like it’s less stigmatised”. (adolescent, female, 15A) “we’ll talk about it she’s overweight. Why not, my God, talk about it so it gets fixed if possibly there is a chance”. (Parent, female, 3B) “Oh yeah, definitely, if I ever feel like it’s getting to a point where I need to lose weight or I feel like I need to change, I’ll speak to my mum and she’ll give me like really good advice”. (adolescent, female, 12A) “I’d always try, I have tried to speak to them about it but like it was kind of like shut down straight away. It was like, you’re skinny, you’re fine”. (adolescent, female, 1A) “I don’t like to bring up weight because I don’t want to cause an issue if there’s not an issue there”. (parent, female, 4B) "I think for parents it’s so painful sometimes and they just don’t know what to do, how to deal with it, so they kind of like go into their denial and hope for the best and I think that’s another issue”. (parent, female, 9B)
3. Nowhere to turn for help	“the place they are meeting is quite far from us and being late in the evening plus I’ve got a three year old and I’m just started driving so, it’s not really an option going there”. (parent, female, 3B) “A professional just saying your child is overweight, need to lose X amount is not going to help me, I need practical suggestions, you know, or recommendations of what I could do”. (parent, female, 8B) “it would be nice to lose the weight but it’s just—I don’t feel anyone has exactly tried to help, like from the NHS side cause sort of what I realised is unless the person has an eating disorder the weight is like they don’t talk about it”. (adolescent, female, 11A) “I have seen like on social media, again, like they’ll tell you things, like how to lose weight and then people will try and it just doesn’t work. Or, it’s just like lies”. (adolescent, female, 12A) “I’d probably look online, just cos although online you can get some misleading information, if you find a happy medium where a lot of websites are telling you a very consistent thing and I’d probably look online”. (adolescent, male, 9A) “I mean to some extent I feel everyone get’s sucked in…even if you’re searching for something healthy like, overnight oats and like you just said, well you can’t just get out, it can always lead to a negative and then suddenly I’ll be on a loophole in that way, hang on, this is not good”. (adolescent, female, 5A)
4. Don’t tell me, show me	“my dad he would support me by going to the gym with me, having meals with me and sitting down with me, talking about how I am feeling and things like that”. (adolescent, female, 7A) “so I try to model that so the boys can see that. OK, these things are really clear and important and I think that kind of motivates them to want to engage in that—sometime they join me in my workouts”. (Parent, female, 2B) “I do try to go to the gym regularly and I do say to her, do you want to come with me, if she does want to come I don’t “you’re coming”, I don’t, you know, if she wants to come with me I do say, oh come on we’ll come together and let’s do this”. (Parent, female, 6B) “I think the worst thing I do is criticise myself in front of her…If I’m picking stuff up that I don’t like about me, and then is she gonna start looking at herself? So, I’ve tried to do that a lot less. But I have done it”. (parent, female, 1B) “I think that it’s hard when parent say, “you need to do this” or “you need to do that” and it seems so much nicer when they say “we’re gonna all do this together, it’s good for all of us”. (adolescent, female, 8A)

NHS, National Health Service.

### ‘Weight pressure as a function of societal norms’

During interviews, parents and adolescents reported there was immense pressure on young people to maintain an ideal body as defined by current societal norms in which ‘thinness’ in females and ‘athletic physiques’ in males are valued. Adolescents told us that while overt weight-based bullying was rare, there was still an unsaid understanding that if you wanted to be liked or popular, you must adhere to societal standards around weight and shape. This pressure was often exacerbated through the portrayal of an ‘ideal body’ on social media. Some adolescents acknowledged the potential benefits of social media in weight control, but many felt that it promoted unhealthy habits and exploited the vulnerabilities of young people around weight and body image.

Although some parents did express concern about the physical consequences of their child’s excess weight, many were much more concerned about the potential mental health repercussions of weight pressures and weight concern in this age group. Their main concerns were their children being upset, ashamed or preoccupied with their weight. The adolescents we spoke to all expressed some degree of weight concern and disordered eating was seen as a way of dealing with weight concern and the pressure to be thin, with some adolescents even describing that they felt pressure from their peers to develop an eating disorder to lose weight and better fit in.

### ‘The parent-child relationship’

All the parents perceived themselves to be overweight and their views and feelings about their own weight appeared to influence how they approached their child’s weight. Parents who felt ashamed or embarrassed about their weight were less likely to discuss weight with their child. Parents relayed that they often felt guilty or blamed themselves for their child’s weight. This guilt and shame often prevented them from discussing weight with their children as they perceived the topic to be too difficult to address. Adolescents were also aware of their parents’ weight and did not want to ‘burden them further’ with their own weight concern. Parents who accepted they were overweight, wanted to tackle it and did not feel ashamed were much more likely to discuss weight with their children, and these conversations were generally normalised as an everyday part of life.

Several teenagers conveyed that they felt comfortable discussing weight, as long as they were the ones who initiated the conversation. They only felt is was appropriate for parents or healthcare professionals to broach the topic of weight if there were health concerns, such as in case study 4. When adolescents did initiate weight discussions with their parents, they mostly sought a listening ear and words of encouragement and became frustrated when they were brushed off with platitudes such as “you’re already perfect just as you are”.

### ‘Nowhere to turn for help’

Adolescents and parents acknowledged the lack of readily available effective support for weight management. Parents believed interventions were not fit for purpose due to barriers such as time, location and conflicting familial priorities and that general practitioners were not helpful in discussing adolescent weight and did not actively offer treatment. One adolescent expressed the view that the only way to receive assistance for weight-related issues was by developing an eating disorder.

Many adolescents said they resorted to the internet or social media for weight loss assistance. The internet was seen as slightly better than social media as they could aim for reputable websites, or look up recipes. They acknowledged that the information provided on social media was often unreliable and ineffective and described it as a ‘rabbit hole’ that could lead to less appropriate material.

### “Don’t tell me, show me”

Some adolescents perceived that their parents supported them in losing weight by engaging in physical activity and healthy eating alongside their children. Even if parents did not discuss weight directly, by role modelling healthy behaviours, adolescents felt motivated and supported.

Several adolescents reported positive experiences such as bonding with their parents over shared activities like family meals, walks or fitness routines. However, some also observed negative behaviours such as parents making negative comments about their own bodies or engaging in unhealthy dieting behaviours. Parents recognised the potential negative effects of their actions on their children and, as a result, worried about transmitting their own body image concerns to their offspring.

### Linked parent-child dyads

Four linked parent-child dyads were interviewed. These are presented as case studies in table 3.

**Table 3:** Linked parent-child dyad case studies

Case study 1: mother/daughter, 16 years, White British Mum felt a lot of shame regarding her own weight and also felt guilty about her children’s weight. She was determined to prevent her children from becoming upset and preoccupied with their weight as she was, so she did not allow them to participate in the National Child Measurement Programme (at age 5 and 11 years) and constantly reassured them that they were perfect and did not need to lose weight. Her daughter felt upset about her mother’s reassurance because she felt that she needed to lose weight and felt that her mother was not responding to her concerns. The daughter had witnessed her mother’s lifelong struggle with weight and did not want to face the same problem, but she also felt that she was not receiving the support she needed.	“she’s watched us going through the pain and probably especially me, cos I’m always moaning about my weight. So, maybe she has watched, but then I suppose… it’s really difficult isn’t it?” (Parent, female, 1B) “my mum she thinks it’s really like a hard topic to touch on. And like they’re always like, oh you so - you’re so thin, like you’re so thin, like, I want to like—I want to not just be thin I want to have like muscle and stuff… I wish they would have been like, okay, let’s look into some gyms that you can go to, or like let’s look at stuff that you can do to start that. Just kind of support me in doing it, instead of shutting the idea down”. (Adolescent, female, 1A)
Case study 2: mother/son, 14 years, Black African Mum stated that, although she considered herself to be overweight, she did not feel shame about her weight. She viewed weight in terms of health and fitness rather than appearance, and normalised conversations around weight with her son. She took practical steps such as encouraging exercise as a family and modelling healthy behaviours to address her and her son’s excess weight. As a result, the son felt comfortable discussing his weight and was motivated to lose weight and improve his health.	“I use myself as an example, sometimes I come out and tell my boys, “see boys I’m adding too much weight, look at my belly, look at my thighs, you know what, the truth is I need to lose weight boys so, what do I need to work on”, that’s what I tell them. So, when we have conversations like that it makes it easier when I approach them to say, “OK, you know when you adding so much, you’re getting bigger” they’re like, “oh that’s true, mum”. Why because I set that pace to make them feel comfortable”. (Mum 2B) “he was receptive cause when I said, OK, you need to lose some weight, you know that, he was like yes, I know mum. You have to make sure you’re healthy, this is not just because I’m saying it, this is also for, for health reasons”. (Mum 2B) “it was very comfortable speaking to my parents and, yeah, like they just listened and that’s basically it”. (Son 2A)
Case study 3: mother/daughter, 16 years, White British Mum was aware that her family tended to be on the ‘larger’ side, and she saw that her daughter took after her. Consequently, starting at a young age, she incorporated healthy habits such as daily exercise and controlled portion sizes into her family’s lifestyle. She frequently mentioned ‘guiding’ her daughter towards good health by setting an example and creating an environment that promoted healthy habits. Despite this, mum refused to acknowledge her daughter’s medical diagnosis of being overweight, which led to frustration in her daughter, who felt that everyone avoided weight discussions. Nevertheless, the daughter felt fully supported by her family in achieving a healthy weight. She was grateful that she had grown up in a household where everyone made a concerted effort to live healthily, which made it easier for her to do the same.	"I think she’s a healthy weight. She would not agree. We are never going to be, you know, really skinny, skinny, people we’re just not them people”. (Mum 6B) “I do try to go to the gym regularly and I do say to her, do you want to come with me, if she does not want to come I don’t go “you’re coming”, I don’t, you know, if she wants to come with me I do say, oh come on we’ll go together and let’s do this”. (Mum 6B) “my mum was very, very helpful. So, she does it with me so that I don’t feel stressed, cos I know that some people when they go to the gym, they feel intimidated”. (Daughter 6A) “throughout my entire life I’ve always seen that my parents have always had healthy foods, going on exercises. We have a dog, so we go on dog walks to keep ourselves exercised. And yeah, I have been brought up into a healthy family”. (Daughter 6A)
Case study 4: mother/daughter, 16 years, White other Mother and daughter were both aware of their overweight status and were actively seeking assistance to manage their weight. However, they felt let down by the medical system, and the mother was concerned about the potential health problems her daughter may face. The daughter recognised the need to lose weight but felt ignored by health professionals who seemed hesitant to address the topic. Both were desperate for support and acknowledged that they could not do it alone, but were frustrated with the lack of resources available to them.	"You know, it doesn’t always have to be a shameful issue, it can be an issue or something of a concern, it can be a conversation topic, absolutely, there is a way”. (Mum 3B) "I know all the theory, I know it all, I even—I did biology and stuff, so my mum is a nurse so I know it all so I don’t need the, you know, this blah-blah stuff, I need the support”. (Mum 3B) "I have hypermobility and having extra weight obviously makes it worse, but instead of telling me like, well you should do these exercises and you should try going to this place or, you know, giving me options. They’re like, well losing weight would be helpful. I’m like, I think, I know that, like how?” (Daughter 11A) "So, like there needs to be like the middle space where like everyone is respected whatever size they are but still being told you’re overweight”. (Daughter 11A) "mental health is like, you know, being given the importance it needs but then other things like physical health in some cases are being like thrown out the window”. (Daughter 11A)

## Discussion

Participants reported that being overweight and their concern about their weight affected their well-being. Societal weight pressures, the parent-child relationship, lack of support from both parents and professionals and the act of role-modelling all had a significant impact on how parents and adolescents perceived weight and weight management.

Adolescents in our study reported that they felt weight pressure, exacerbated by social media, to which body dissatisfaction was attributed. This finding has been reported previously, with literature citing a relationship between increased social media use and body dissatisfaction^17^,^20^ and, in some cases, higher depressive symptom scores.^21 22^ Parents in our study were deeply concerned about the pressure young people face regarding their weight and how this could impact their mental health. This concern was especially strong in parents of female adolescents, whereas we and others have found that body dissatisfaction was similar across genders.^17^ Adolescents in our study perceived that being above a healthy weight meant that they were excluded from certain peer groups, which was their primary motivation for weight loss. This contrasts with a narrative review of qualitative studies in which adolescents reported that health was their primary motivator for weight loss, and improved body image was secondary.^23^ Although the evidence suggests that weight loss should be encouraged for improved health, if societal pressure is the primary motivator this could lead to increased weight-based stigma and suggests that perhaps an alternative narrative that encourages body positivity and emphasises shifting societal norms around weight, should be sought.

All the parent participants in our study were themselves above a healthy weight and we found that parental feelings towards their own weight seemed to influence how they approached their child’s weight. As with previous research,^24^ many of the parents expressed guilt or felt blame for their child’s weight, and this was seemingly magnified if the parent felt shame or guilt about their own weight. A previous study of parent/adolescent body shame showed that child body shame was positively correlated with parental body dissatisfaction in linked parent-child dyads.^25^ In our linked dyads where parents demonstrated body dissatisfaction, children did as well. We found that parents who had experienced weight stigma were more likely to seek professional help to lose weight, a finding that is mirrored in the literature.^26^

English (National Institute for Health and Care Excellence) healthcare guidance states that healthcare professionals and those working with children should take every opportunity to raise the issue of weight with families and refer to weight management interventions.^4^ However, adolescents interviewed for this study wanted to initiate weight management conversations on their own terms. They wanted support to be available but only when they sought it, not when someone else, whether it be parents or healthcare professionals, told them they needed it. Adolescents spoke of shame or embarrassment if others initiated weight-based conversations, and indeed, literature in this area shows that adolescents above a healthy weight, compared with healthy-weight adolescents, are more likely to become upset when the topic of weight is raised by a parent or carer.^27^ The exception to this was when there were weight-related health concerns, such as in case study 4, on which participants felt the topic could be addressed. This suggests that, counter to national guidance, adolescents may not welcome these discussions if healthcare professionals were to follow the guidance, but none of our participants reported experience of this happening. Our findings suggest a need for further research on how professionals might allow adolescents to drive the conversations and to be equipped to offer routes for support if these conversations arise.

### Strengths and limitations

This study had some limitations. While we enrolled a diverse range of ethnicities, ages and deprivation levels, parental gender was limited to females, and the majority of the young people were also female. Therefore, the results may not be applicable to the wider population, particularly males. Additionally, by having participants self-report their weight status, there is no guarantee that they were actually above a healthy weight. The decision to accept non-linked dyads meant that specific parent-child dynamics were limited. Furthermore, individuals who have previously sought weight management or have higher concerns about their weight may have been more inclined to participate in this study, which could bias the results. Additionally, the convenience and snowball sampling approaches may mean less variation in demographics and therefore less variance in opinion. Obtaining qualitative data from adolescents is difficult, especially about sensitive topics, such as weight^25^ and this, combined with the power differential between the interviewee and the adolescent participants, may have meant they withheld crucial information. At the time of conducting this research, glucagon-like peptide 1 agonists were not approved for use in adolescents and, therefore, were not covered in these interviews. However, this could be a potential avenue for further research.

## Conclusions

Our findings suggest a complex dynamic between adolescents who are overweight and their parents. Weight concern, encompassing mental, physical and psychosocial health, was pervasive among both parents and adolescents. For young people of both genders, societal pressure was the primary motivator for weight loss. Parents’ feelings about their own weight were highly influential on both body image concern and help-seeking behaviour among adolescents, suggesting that an improvement in parental weight concern could lead to improvements in adolescent concern as well. Counter to national guidance, our research suggests that adolescents do not want parents or healthcare professionals to initiate weight discussions unless there are health concerns. Instead, they suggest role-modelling as an acceptable alternative to initiating healthy behaviour change within the parent-child relationship.

## Supplementary material

10.1136/archdischild-2024-327306 online supplemental appendix 1

## Data Availability

Data are available on reasonable request.
