# Supplementary material for: Adolescent and parental views on weight and weight management: a qualitative study
Source: Arch Dis Child. 2025 Feb 6;110(8):e327306. doi: 10.1136/archdischild-2024-327306 (PMC12320593; doi:10.1136/archdischild-2024-327306)
Supplement: online supplemental appendix 1 [file archdischild-110-8-s001.pdf]

<u>Interview Guide</u>

1. Interviews with <u>parents</u> of young people above a healthy weight

I. Feelings towards your child's weight

Do you feel that your child is overweight?

a. *Probe 1: Are you aware of how excess weight in children is measured or any clinical risks associated with being overweight?*
b. *Probe 2: Do you think your child would like to lose weight?*
c. *Probe 3: Have you or your child ever experienced any stigma or bullying based on their weight? How does this make you feel?*
d. *Probe 4: Do you feel like your own weight effects your feelings about your child's weight?*
e. *Probe 5: Do you think there has been anyone or anything has as strong influence on your child's weight?*

II. The effect of weight on the parent/child relationship

What effect, if any, do you feel that your child's weight has on your relationship with your child?

a. *Probe 1: Have you ever discussed your child's weight with them? If so, how did that conversation go?*
b. *Probe 2: Do you feel like your child would be receptive to you discussing weight with them?*
c. *Probe 3: What, if any, effect do you feel these discussions would have on your child's self-esteem or mental health? How do you think you could minimise these effects?*
d. *Probe 4: Do you feel like it is your role as a parent to support your child with their weight? If not, whose role is it?*

III. Your child's weight management journey

Can you tell me about anything you as a family, or your child, has done, that you are aware of, to control their weight?

a. *Probe 1: What are your feelings on any weight control behaviours your child may have engaged in?*
b. *Probe 2: Do you feel there is anything that you, or anyone else, could do to better support your child with their weight?*
c. *Probe 3: To what extent do you feel your child's school or environment effect their ability to lose weight?*

IV. Summary

We’ve now reached the end of the interview. Is there anything that was not mentioned in today’s discussion that you would like to add or would you like to further discuss any issue that was previously raised?

2. Interviews with young people above a healthy weight

I. Feelings towards your weight

Do you feel that you are overweight?

a. *Probe 1: What is your reasons for labelling yourself overweight?*
b. *Probe 2: Do you want to lose weight? If so, what are your main motivations for losing weight?*
c. *Probe 3: Have you ever experienced any stigma or bullying based on your weight? If so, how did that make you feel?*
d. *Probe 4: What or who do you feel is responsible for your excess weight?*

II. The effect of weight on the parent/child relationship

What effect, if any, do you feel that your weight has on your relationship with your parent(s)?

e. *Probe 1: Have you ever discussed your weight with your parent(s)? If so, how do those conversations go? How would they/do they make you feel?*
f. *Probe 2: Would you be ok if your parents brought up your weight in conversation?*
g. *Probe 3: What, if any, effect do you feel these conversations (would) have on your self-esteem or mental health? How could these effects be minimised?*
h. *Probe 4: Do you feel that your parents should play a role in your weight loss?*

III. Your weight management journey

Can you tell me about anything you, or your family, have done, to help control your weight?

d. *Probe 1: Do you feel supported in any weight control behaviours you may take part in?*
e. *Probe 2: Do you feel there is anything that your parents could do to better support you with your weight?*
f. *Probe 3: To what extent do you feel your school, environment or peers effect your ability to lose weight?*

IV. Summary

We’ve now reached the end of the interview. Is there anything that was not mentioned in today’s discussion that you would like to add or would you like to further discuss any issue that was previously raised?
